# Development and experimental verification of a prognosis model for disulfidptosis-associated genes in HNSCC

**DOI:** 10.1097/MD.0000000000037308

**Published:** 2024-03-22

**Authors:** Yushen Li, Lu Tao, Jiajun Xin, Yifei Dai, Xiantao Chen, Jiatong Zou, Rui Wang, Bowei Wang, Zhihui Liu

**Affiliations:** a Hospital of Stomatology, Jilin University, Changchun, People’s Republic of China; b Jilin Provincial Key Laboratory of Tooth Development and Bone Remodeling, Changchun, People’s Republic of China; c The Second Hospital of Jilin University, Changchun, People’s Republic of China.

**Keywords:** disulfidptosis, head and neck squamous cell carcinoma, survival prognosis, tumor microenvironment

## Abstract

Disulfidptosis is a newly discovered cell death pattern that has been less studied in head and neck squamous carcinoma (HNSCC). Exploring the molecular features of different subtypes of HNSCC based on disulfidptosis-associated genes (DAGs) is important for HNSCC. In addition, immunotherapy plays a pivotal role in the treatment of HNSCC. Exploring the sensitivity of immunotherapies and developing predictive models is essential for HNSCC. We analyzed the expression and mutational status of DAGs in 790 HNSCC patients and correlated the dates with clinical prognosis. HNSCC patients were divided into 2 groups based on their DAG expression. The relationship between DAGs, risk genes, and the immune microenvironment was analyzed using the CIBERSORT algorithm. A disulfidptosis risk model was constructed based on 5 risk genes using the LASSO COX method. To facilitate the clinical applicability of the proposed risk model, we constructed column line plots and performed stem cell correlation analysis and antitumor drug sensitivity analysis. Two different disulfidptosis-associated clusters were identified using consistent unsupervised clustering analysis. Correlations between multilayer DAG alterations and clinical characteristics and prognosis were observed. Then, a well-performing disulfidptosis-associated risk model (DAG score) was developed to predict the prognosis of HNSCC patients. We divided patients into high-risk and low-risk groups based on the DAG score and found that patients in the low-risk group were more likely to survive than those in the high-risk group (*P* < .05). A high DAG score implies higher immune cell infiltration and increased mutational burden. Also, univariate and multivariate Cox regression analyses revealed that the DAG score was an independent prognostic predictor for patients with HNSCC. Subsequently, a highly accurate predictive model was developed to facilitate the clinical application of DAG scores, showing good predictive and calibration power. Overall, we present a comprehensive overview of the DAG profile in HNSCC and develop a new risk model for the therapeutic status and prognosis of patients with HNSCC. Our findings highlight the potential clinical significance of DAG and suggest that disulfidptosis may be a potential therapeutic target for patients with HNSCC.

## 1. Introduction

Head and neck cancer (HNC) ranked sixth among all cancer types worldwide and includes cancers originating from the lips, mouth, nasopharynx, larynx, hypopharynx, etc.^[1,2]^ According to GLOBOCAN data compiled by the International Agency for Research on Cancer in 2020, the global incidence of HNC is about 870,000 and the global death toll from HNC is about 440,000,^[3]^ which indicates that more attention should be paid to the huge burden caused by HNC.

Head and neck squamous cell carcinoma (HNSCC), the most common type of HNC pathology, accounts for approximately 90% of HNC cases. In the past decades, despite significant improvements in strategies for treating HNSCC such as surgery, radiotherapy, chemotherapy, and immunotherapy, the overall survival and quality of life of HNSCC have not improved accordingly.^[4]^ Therefore, there is an urgent need to further explore the mechanisms of head and neck squamous cell carcinoma development and to screen for effective biomarkers to predict the early diagnosis and long-term prognosis of head and neck squamous cell carcinoma.^[5]^

Abnormal accumulation of intracellular disulfides, such as cystine, induces disulfide stress and is highly toxic to cells.^[6,7]^ A reduced form of nicotinamide adenine dinucleotide phosphate (NADPH) provides critical reducing capacity to counter disulfide stress and maintain cell survival. The NADPH pool in the cell membrane is produced mainly from glucose via the pentose phosphate pathway. In cancer cells with abnormal expression of the cysteine transporter solute carrier family 7 member 11 (SLC7A11), upon binding to glucose starvation, cysteine uptake is high and cysteine is reduced to cysteine, depleting the NADPH pool and leading to the accumulation of large intracellular disulfide molecules and rapid cell death.^[6,7]^ However, the nature of this cell death remains unknown. In this^[8]^ study, the mechanism of this cell death was elucidated: high expression of SLC7A11 combined with glucose starvation induces a hitherto unrecognized regulated cell death that is induced by disulfide stress, independent of ATP depletion or cystine crystal formation, defining this cell death as disulfidptosis. Therefore, the potential role and mechanisms of disulfide deposition in the development of HNSCC require further investigation.

In this study, we evaluated the expression profile of disulfidptosis -associated genes (DAG) and analyzed their relationship with tumor metabolism and immune microenvironment. A disulfidptosis risk model was developed to predict the prognosis of HNSCC patients.

## 2. Methods

### 2.1. Datasets and preprocessing

The HNSCC dataset was downloaded from the Gene Expression Omnibus (www.ncbi.nlm.nih.gov/geo/) and The Cancer Genome Atlas (TCGA) (portal.gdc.cancer.gov) databases. The Gene Expression Omnibus HNSCC cohort GSE65858 and TCGA cohort were analyzed using R (version 4.1.3) and the R Bioconductor package.

### 2.2. Identification of differentially expressed DAGs

Fourteen DAGs were obtained from previous studies. differences in DAG expression in HNSCC and normal tissues were analyzed using the “limma” and “reshape2” packages.

### 2.3. Mutation analysis

Mutation frequencies and oncoplot waterfalls of DAG and risk genes in HNSCC patients were generated by the “maftools” package. The “rcircos” package in R was used to map CNV changes in 14 DAGs on 23 chromosomes Location

### 2.4. Consensus clustering analysis of DAGs

The R package “ConsensusClusterplus” is used for unsupervised consensus clustering analysis to classify patients into different molecular subtypes based on DAG expressions. For clustering, the cumulative distribution function curve increases smoothly and all subtypes are represented by a sufficient number of samples.

### 2.5. Molecular expression patterns and clinical features

We compared the relationship between HNSCC stage, age, sex, node stage, tumor stage, and prognosis to analyze their clinical value.

### 2.6. Functional enrichment analysis

Gene Ontology and the Kyoto Encyclopedia of Genes and Genomes (KEGG) were performed using the “ggplot2” package. Gene set variation analysis (GSVA) was performed using the flagged gene set (c2.cp.kegg.v7.2) from the MSigDB database.

### 2.7. Disulfidptosis risk model

The “limma” package was used to analyze the differentially expressed genes (DEGs) between different disulfidptosis subtypes with a multiplicative change of 1.5 and an adjusted *P* value < .05. Cox regression analysis was used to assess the prognostic value of DEGs. A DAG risk model (disulfidptosis risk model) was constructed based on DEGs using LASSO Cox regression analysis.

All HNSCC patients were randomly assigned to the training group (n = 395) and the test group (n = 394), and a prognostic risk score associated with disulfidptosis was constructed using the training group. In the training group, patients were divided into low- and high-risk subgroups based on the median risk score, and overall survival (OS) time was compared between the 2 subgroups using Kaplan–Meier analysis. The DEG-based principal components analysis was performed using the “prcomp” function in the “stats” package. In the test study, the expression of each DEG associated with disulfidptosis was also normalized by the “proportion” function. The test set was used to validate the model.

### 2.8. Prognosis of squamous carcinoma of the head and neck

OS was assessed by the “survival” and “survminer” packages.

### 2.9. Correlation of HNSCC disulfidptosis Risk Model with the tumor microenvironment (TME)

The ESTIMATE algorithm was used to assess immune scores. We used the CIBERSORT algorithm to analyze the scores of 23 human immune cell subpopulations in each HNSCC sample. The level of immune cell infiltration in HNSCC TME was analyzed using the single sample gene set enrichment analysis algorithm. Single sample gene set enrichment analysis was used to quantify the abundance of 23 infiltrating immune cells in heterogeneous samples.

### 2.10. Clinical correlation and stratification analysis of the disulfidptosis risk model

The relationship between the disulfidptosis risk model and clinical factors such as age, gender, and HNSCC stage was analyzed using chi-square tests and Wilcoxon rank sum tests.

### 2.11. Mutation, drug sensitivity analysis, and cancer stem cell index in HNSCC

The “maftools” package is used to analyze somatic mutations in HNSCC. The “pRRophetic” package is used to calculate the semi-inhibitory concentration (IC_50_) values of commonly used chemotherapeutic agents against HNSCC. We analyzed the Spearman correlation between the abundance of immune cells and DAG scores using R.

### 2.12. Construction and validation of column line graphs and scoring systems

The construction of predictive line graphs requires the use of the “rms” package. Column plots are evaluated using time-dependent receiver operating characteristic curves. Cox risk analysis was used to identify independent risk factors for OS.

### 2.13. Quantitative real-time PCR analysis

Four cancerous tissues and 4 normal tissues were collected from the Hospital of Stomatology, Jilin University. TRIeasy Total RNA Extraction reagent (Yeasen Biotech, Shanghai, China) was added to the tissue and homogenized with a homogenizer (JXFSTPRP-24, Shanghai Jingxin Industrial Development Co., Ltd., Shanghai, China) using the following parameters: 3 grinding times, a frequency of 73 Hz, an interruption time of 3 s, and a running time of 60 s. The cDNA was synthesized using the Hifair III 1st strand cDNA Synthesis SuperMix for quantitative real-time PCR (Yeasen Biotech). The primer sequences used are shown in Table 1.

**Table 1:** Primer sets used for qPCR.

Genes	Primer Sequence, 5′–3′	
	Forward	Reverse
FAM83E	TTCAGCCTTGAGTTCCGGAC	AGAATGTCACTGAGTGCCGG
EFEMP1	ACCTTTTCCATTTTCTTACAAAGC	ATGATCTTCTGTGGTGCTTAAGG
CD79A	CAAGAACCGAATCATCACAGCC	TCTGCCATCGTTTCCTGAACA
DKK1	TTCCAAGAGATCCTTGCGTT	ACCCCATTGATTGTTATCTTGA
SPINK7	CCTGCCCCATCACATACCTA	AGAGCCTGGGATGATGAAGATG
GAPDH	CAACAGCCTCAAGATCATCA	CCATCACGCCACAGTTTC

### 2.14. Statistical analysis

To assess the independent prognostic value of the risk model, Cox regression models were used in the study. All statistical analyses were performed using R version 4.1.3. Statistical significance was set at *P* < .05.

## 3. Results

### 3.1. Genetic variation of disulfidptosis- associated genes in HNSCC

**Figure 1.**
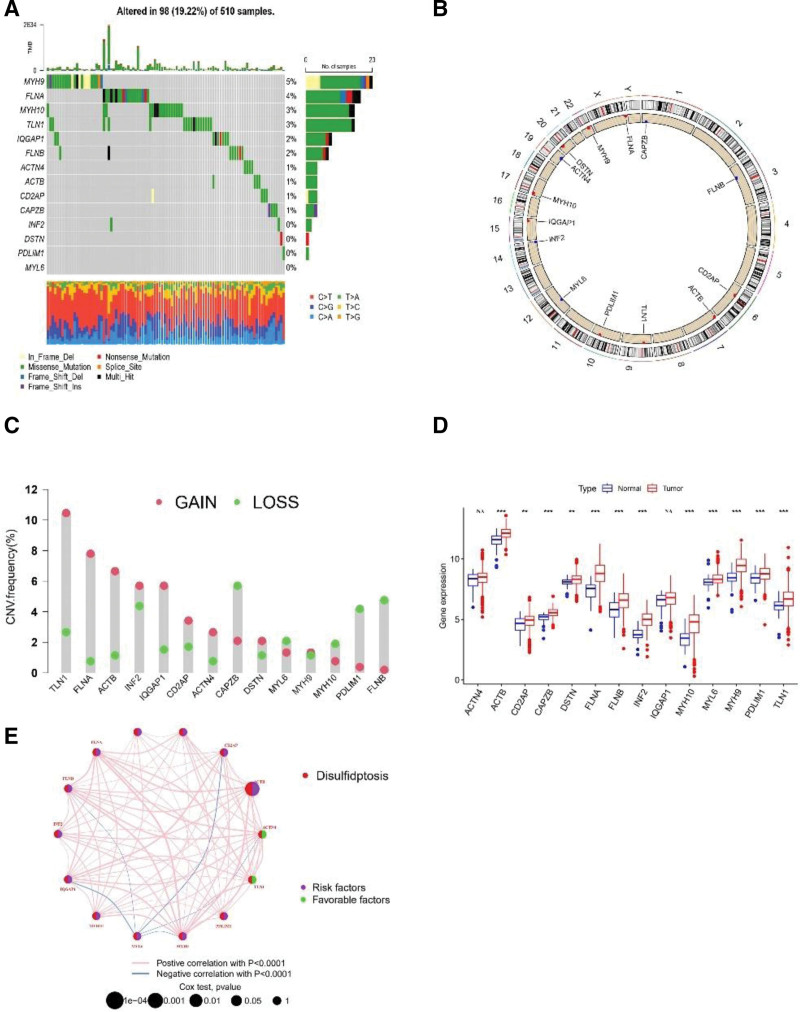
Genetic variation of disulfidptosis apoptosis-related genes in HNSCC. (A) Mutation frequency and classification of 14 DAG in HNSCC. (B) Mutation frequency and classification of 14 DAGs in HNSCC. (C) CNV variant frequencies of 14 DAGs in HNSCC. The height of the column represents the frequency of change. (D) 14 DAGs expression differentially in HNSCC and normal tissue, Tumor, red; normal, blue. *P* values were showed as: *, *P* < .05; **, *P* < .01; ***, *P* < .001; NA, no difference in statistics. (E) A network describes the connection and prognostic values of 14 disulfidptosis -associated genes. DAGs = disulfidptosis-associated genes, HNSCC = head and neck squamous carcinoma.

A total of 14 genes associated with disulfidptosis were obtained based on previous studies,^[8]^ which were named disulfidptosis-associated genes (DAGs) in this study. Of the 510 HNSCC patients in the TCGA cohort, 98 (19.22%) had gene mutations, with the highest frequency of MYH9 mutations (5%), followed by FLNA, MYH10and TLN1(Fig. 1A). We investigated the CNV frequencies of 14 DAGs in the HNSCC. TLN1 exhibited the highest amplification frequency, while CAPZB, FLNB, and PDLIM1 were lost at a high frequency (Fig. 1C). Figure 1B shows the location of CNV alterations in 14 DAGs on chromosome 23. Then, we explored the expression levels of 14 DAGs. 12 DAGs were upregulated in tumor samples, including ACTB, CD2AP, CAPZB, DSTN, FLNA, FLNB, INF2, MYH10, MYL6, MYH9, PDLIM1, and TLN1(Fig. 1D).

We first show the connections and prognostic values for the 14 DAGs in Figure 1E.

### 3.2. Identification of gene clusters associated with disulfidptosis in HNSCC

**Figure 2.**
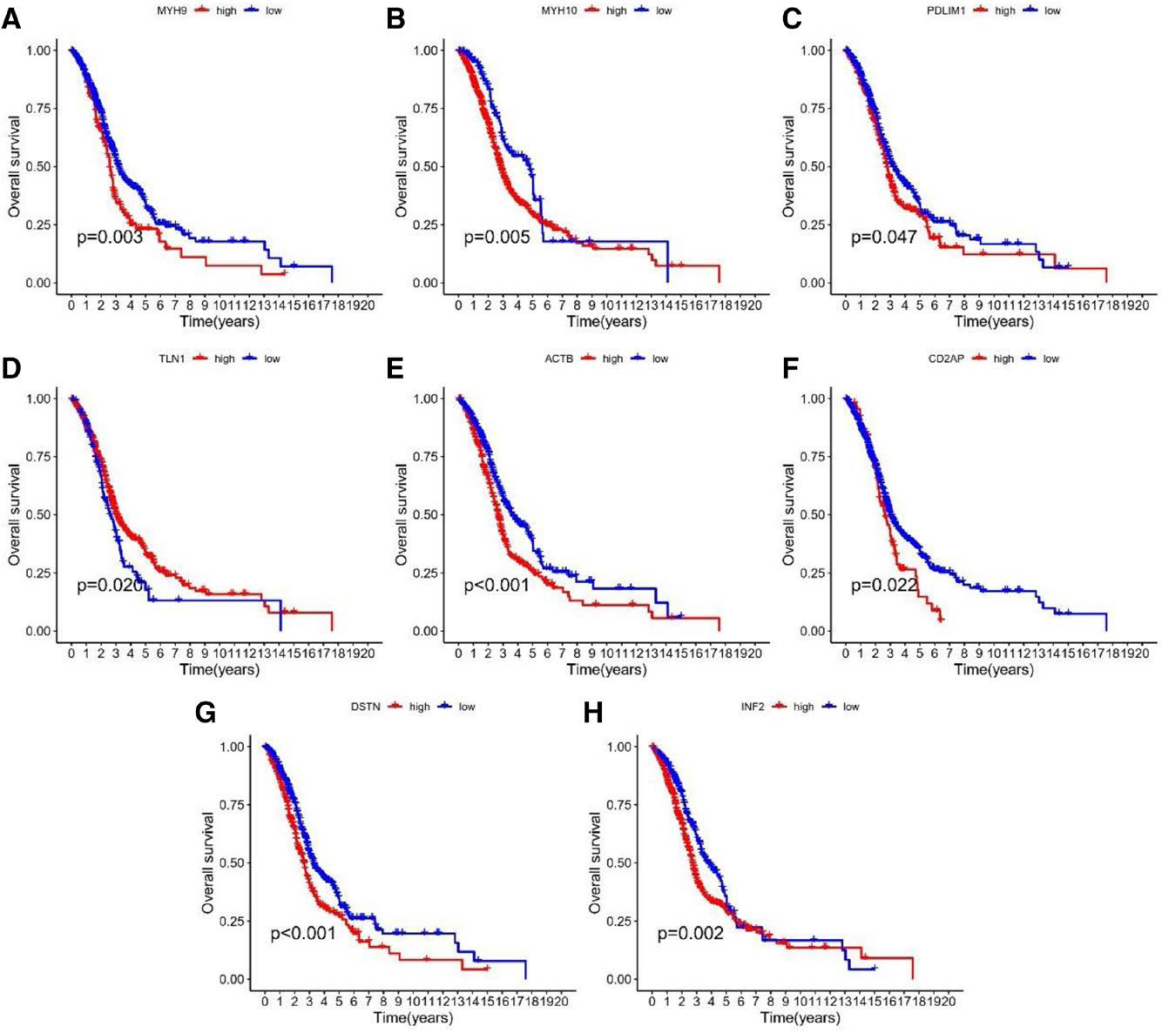
Kaplan–Meier curves of 14 disulfidptosis -associated genes in TCGA cohort. TCGA = The Cancer Genome Atlas.

To gain a clear understanding of the correlation between DAG and clinical prognosis, we used Kaplan–Meier analysis and univariate Cox regression analysis to identify 8 prognostic DAGs (Fig. 2A–H).

The results of multivariate Cox regression analysis further revealed that one prognostic DAG (ACTB) was an independent prognostic factor (Table 2).

**Table 2:** Multivariate cox regression analysis of 14 disulfidptosis-associated genes associated with overall survival in patients with HNSCC.

Gene	HR (95% CI)	*P* value
ACTN4	0.982 (0.833–1.157)	.829
ACTB	1.522 (1.202–1.928)	.0005
CD2AP	1.091 (0.916–1.300)	.328
CAPZB	1.043 (0.778–1.398)	.779
DSTN	1.231 (0.996–1.522)	.055
FLNA	1.093 (0.968–1.235)	.152
FLNB	1.010 (0.883–1.155)	.885
INF2	1.195 (0.994–1.437)	.058
IQGAP1	1.081 (0.925–1.263)	.327
MYH10	1.091 (0.989–1.203)	.081
MYL6	1.104 (0.886–1.377)	.378
MYH9	1.073 (0.929–1.240)	.338
PDLIM1	1.120 (0.943–1.329)	.196
TLN1	0.956 (0.839–1.089)	.498

**Figure 3.**
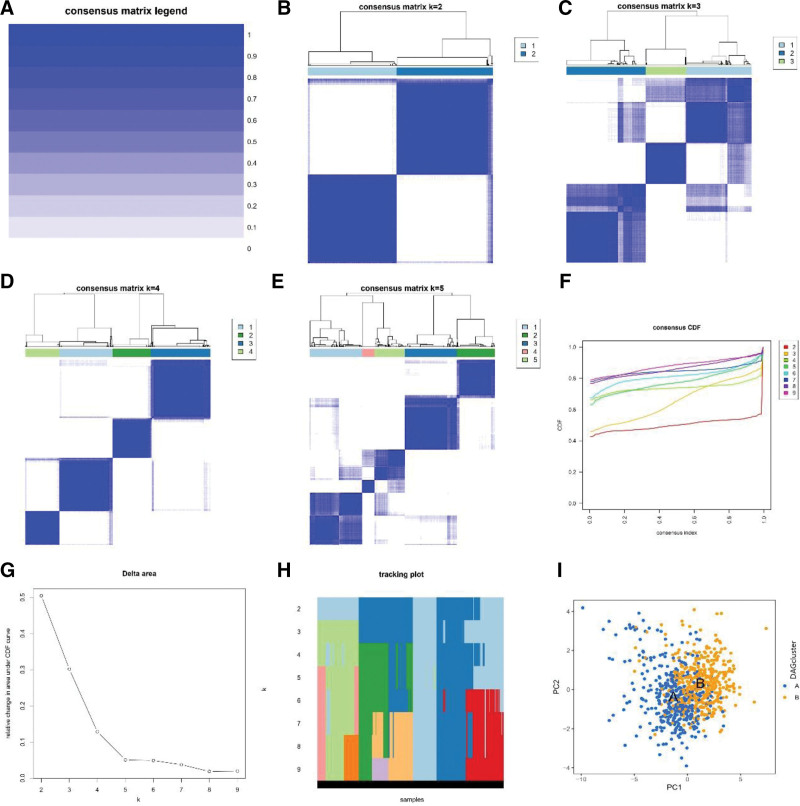
Unsupervised clustering for DAGs. (A–E) TCGA-HNSCC cohort was grouped into 2 clusters according to the consensus clustering matrix (k = 2). (F) Uniform clustering CDF with k from 2 to 9. (G) The change of area under CDF curve with k from 2 to 9. (H) The tracking plot showed the relationship between samples and clusters. (I) The PCA plot showed the distribution of samples among 2 DAG clusters. CDF = cumulative distribution function, DAGs = disulfidptosis -associated genes, HNSCC = head and neck squamous cell carcinoma, PCA = principal component analysis, TCGA = the Cancer Genome Atlas.

To investigate the expression characteristics and potential biological features of DAGs in HNSCC, HNSCC patients in the TCGA-HNSCC and GSE65858 cohort were classified using a consensus clustering algorithm. Based on the expression of 14 DAGs, patients were classified into DAG cluster A (n = 379) and DAG cluster B (n = 411) (Fig. 3A–H). Principal components analysis plots indicated a distinctly different distribution among DAG clusters (Fig. 3I).

### 3.3. Correlation of disulfidptosis -related gene clusters with clinical features and infiltrating immune cells

**Figure 4.**
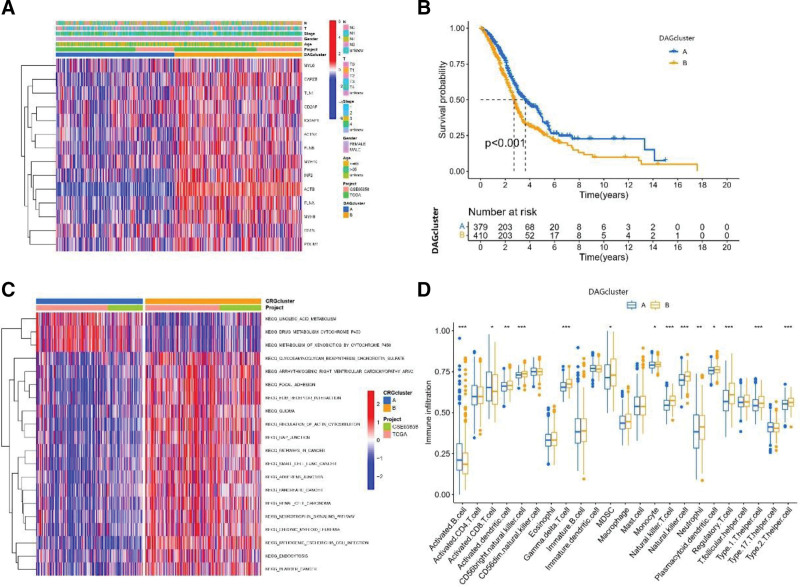
Correlation of disulfidptosis-related gene clusters with clinical features and infiltrating immune cells. (A) The heatmap showed the different expressions of DAGs and clinicopathological characteristics between DAG cluster A and B. (B) Landmark survival analysis for 2 DAG clusters. The overall survival probability of HNSCC patients in the 2 DAG clusters was calculated by Kaplan–Meier analysis (log-rank tests). (C) GSVA of Kyoto Encyclopedia of Genes and Genomes (KEGG) terms between DAG cluster A and B, in which red and blue represent activated and inhibited, respectively. (D) Differences in the abundance of infiltrating immune cells between DAG cluster A and B (DAG cluster A, blue; DAG cluster B, red) *P* values were shown as: *P < .05; **P < .01; ***P < .001. DAGs = disulfidptosis -associated genes, HNSCC = head and neck squamous cell carcinoma.

Figure 4A shows the different expression and clinicopathological characteristics of DAGs between DAG clusters A and B. The expression of 14 DAGs was upregulated in DAG cluster B compared to A. Kaplan–Meier curves showed that patients in group B had better OS than those in group A (Fig. 4B). To further explore the functional annotation between DAG clusters A and B, GSVA was performed. The GSVA of KEGG terms showed that DAG cluster A was abundant in metabolism-related pathways (linoleic acid metabolism, drug metabolism cytochrome p450, metabolism of xenobiotics by cytochrome p450). Cluster B is highly expressed in cancer-related pathways (pathways in cancer, small cell lung cancer, pancreatic cancer, renal cell carcinoma, and bladder cancer), cellular process pathways (focal adhesion, gap junction, adherens junction, endocytosis, and regulation of actin cytoskeleton) and other disease pathways (glioma, pathogenic Escherichia coli infection, pathogenic escherichia coli infection, and arrhythmogenic right ventricular cardiomyopathy arvc) (Fig. 4C). We observed that activated B cells, activated CD4 T cells, and monocyte were significantly higher in DAG cluster A than in DAG cluster B. In contrast, activated dendritic cell, CD56 bright natural killer cell, gamma delta T cell, MDSC, natural killer T cell, natural killer cell, neutrophil, plasmacytoid dendritic cell, regulatory T cell, type 1 T helper cell, and type 2 T helper cell were lower in DAG cluster A than in DAG cluster B (Fig. 4D).

### 3.4. Comprehensive analysis of HNSCC gene clusters based on DEGs

**Figure 5.**
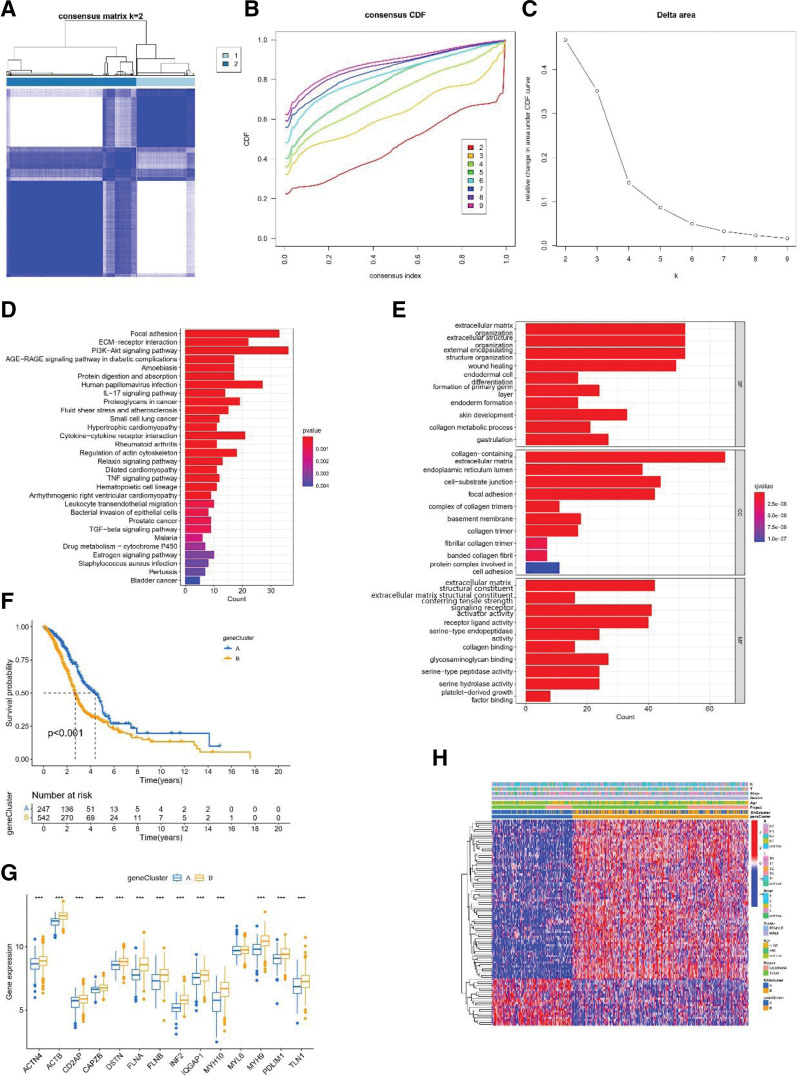
Comprehensive analysis of gene subtypes based on DAGs in HNSCC. (A, C) Cluster analysis of DAGs identified 2 gene clusters. (D, E) GO and KEGG enrichment analysis of DAGs in 2 gene clusters. (F) Survival analysis of 2 Gene clusters. (G) Unsupervised cluster analysis of DAGs. Association between 2 gene clusters and clinicopathological features. (H) Differential expression of 14 disulfidptosis genes in 2 gene clusters. DAGs = disulfidptosis-associated genes, GO = Gene Ontology, HNSCC = head and neck squamous cell carcinoma, KEGG = Kyoto Encyclopedia of Genes and Genomes.

We identified 398 differentially expressed genes (DEGs) associated with disulfidptosis clusters using the “limma” R package. Using univariate Cox regression analysis, 96 cluster-related DEGs were identified to be associated with OS (*P* < .05) for subsequent analysis. Clustering analysis was performed and the results identified 2 gene clusters, referred to as gene clusters A and B. Based on DEG levels, the 2 gene clusters could be distinguished (Fig. 5A–C). To elucidate the function of DEGs, pathways were analyzed using Gene Ontology and KEGG databases. We found that most of the identified DEGs contribute to the regulation of the tumor metabolic microenvironment (Fig. 5D). KEGG pathway analysis showed that the main pathways involved in DEGs include signal transduction, cellular processes, human papillomavirus infection, etc. (Fig. 5E). Kaplan–Meier curves showed that patients in group A had better OS than those in group B (Fig. 5F).

Based on gene expression data, the expression of the 14 DAGs, except MYL6, differed significantly between the 2 gene clusters for the remaining 13 genes (Fig. 5G). In addition, the heatmap shows significant differences in clinicopathological characteristics between the different subtypes (Fig. 5H).

### 3.5. Independent prognostic analysis and development of the disulfidptosis model

To determine whether DAG scores could be used as an independent prognostic predictor of OS, we performed univariate and multivariate Cox regression analyses incorporating and DAG scores showed significant differences in all patients group (Table 3).

**Table 3:** Univariate and multivariate analysis of overall survival (OS) for prognostic parameters in all patient groups.

Variable	Univariate analysis HR (95% CI)	*P* value	Multivariate analysis HR (95% CI)	*P* value
Riskscore	1.984 (1.549–2.542)	5.68E-08	1.952 (1.526–2.497)	9.88E-08
N		.007		.010
N (1)	1.000 (0.711–1.407)	.999	0.793 (0.631–0.995)	.045
N (2)	1.455 (1.149–1.843)	.002	0.799 (0.605–1.056)	.114
N (3)	1.524 (0.774–3.003)	.223	1.125 (0.903–1.400)	.293

**Figure 6.**
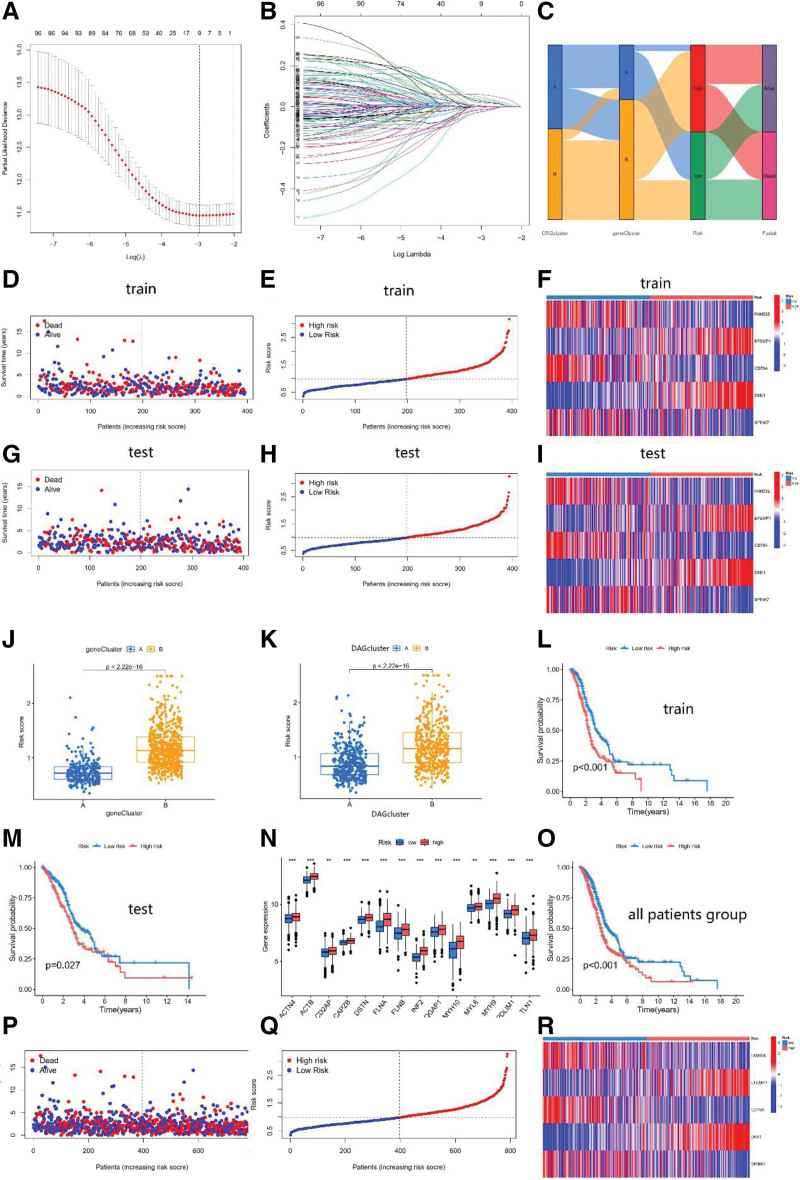
Development of the disulfidptosis model. (A, B) LASSO regression determined the optimal risk genes. (C) Sankey diagram showed the changes in DAG clusters, gene clusters, and status. (D–I) In the training group and testing group, Ranked point and scatter plots of disulfidptosis risk score distribution and patient survival status. Expression and distribution of risk genes. (J) Differences in disulfidptosis risk scores among the 2 gene clusters. (K) Differences in disulfidptosis risk scores among the 2 DAG clusters. (L–M) Kaplan–Meier curve was used to analyze the survival rate of patients with high or low disulfidptosis risk scores in the training group and testing group. (N) The expression of risk genes in high and low-risk groups. (O) Kaplan–Meier curves were used to analyze the survival of patients with high and low disulfidptosis risk scores in all patients group. (P-R) Ranking points and scatter plots of disulfidptosis risk score distribution and patient survival status in all patient groups. Expression and distribution of risk genes. *P* values were showed as: *, *P* < .05; **, *P* < .01; ***, *P* < .001. DAGs = disulfidptosis-associated genes.

The disulfidptosis model was developed based on gene cluster-associated DEGs. LASSO regression was used to determine the optimal λ value, and 9 risk genes were identified (Fig. 6A and B). We then performed multivariate Cox regression analysis for the 9 OS-associated risk genes based on the Akaike information criterion (AIC) values and obtained 5 risk genes (FAM83E, EFEMP1, CD79A, DKK1, and SPINK7). The disulfidptosis model was constructed as follows: disulfidptosis risk score = Exp (FAM83E) × (−0.0878) + Exp (EFEMP1) × (0.1012) + Exp (CD79A) × (−0.0667) + Exp (DKK1) × (0.1182) + Exp (SPINK7) × (−0.0470). Patients with a lower than median risk of disulfidptosis were assigned to the low subgroup, while the other patients were assigned to the high subgroup. Sankey plots show the distribution of patients in the 2 DAG clusters, the 2 gene clusters, the high subgroup or the low subgroup, and the corresponding survival status (Fig. 6C). In both the training group (Fig. 6D–F) and the testing group (Fig. 6G–I), the risk distribution plots of the disulfidptosis show that as the disulfidptosis risk score increases, patients have an increased risk of death and a shorter survival time. In addition, analysis of risk gene expression using the disulfidptosis risk score showed that EFEMP1 and DKK1 were high risk genes, while FAM83E, CD79A, and SPINK7 were low-risk genes. Among the gene clusters (Fig. 6J), cluster A was associated with a low disulfidptosis risk score, while cluster B was associated with a high score. In the DAG cluster (Fig. 6K), cluster A was associated with a low disulfidptosis risk score. DAG cluster B was significantly associated with a higher score. In both the training group (Fig. 6L) and the test group (Fig. 6M), patients with high disulfidptosis risk scores had lower survival rates than those with low scores, with Kaplan–Meier survival curves showing significant differences. In addition, the expression of 14 DAG genes differed between the 2 groups (Fig. 6N). We included all patients in the new test group to verify the correlation between risk score and survival time. The risk of patient death increased together with the disulfidptosis risk score, with a corresponding decrease in survival time (Fig. 6P–R). In addition, the Kaplan–Meier survival curve (Fig. 6O) showed that patients with a high disulfidptosis risk score had a lower survival rate than those with a low score.

### 3.6. Tumor microenvironment between high-risk and low-risk populations

**Figure 7.**
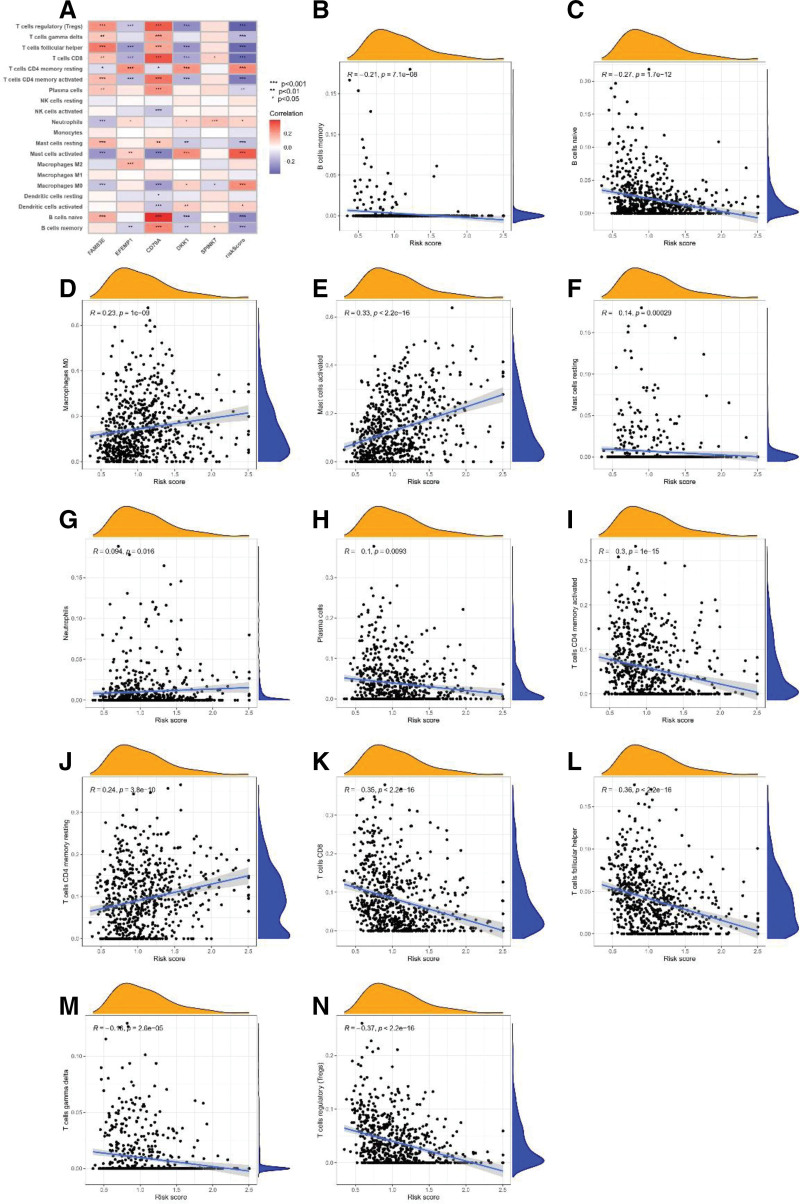
Tumor microenvironment between high-risk and low-risk populations. (A) Correlations between the abundance of immune cells and 5 genes in the disulfidptosis-related prognostic model. (B-N) Correlations between the abundance of immune cells and the DAG score. DAGs = disulfidptosis-associated genes, HNSCC = head and neck squamous cell carcinoma.

Most immune cells were significantly associated with these 5 risk genes (Fig. 7A). As shown in Figure 7B–N, the disulfidptosis risk score was positively correlated with macrophage M0, mast cells activated, neutrophils, and T cells CD4^+^ memory resting. In contrast, there was a negative correlation with T cells gamma delta, T cells follicular helper, CD8^+^ T cell, T cells CD4^+^ memory activated, plasma cells, mast cells resting, B cells naive, B cell memory, and T regulatory cells (Tregs).

### 3.7. Mutation, drug sensitivity, analysis, and cancer stem cell index in HNSCC

**Figure 8.**
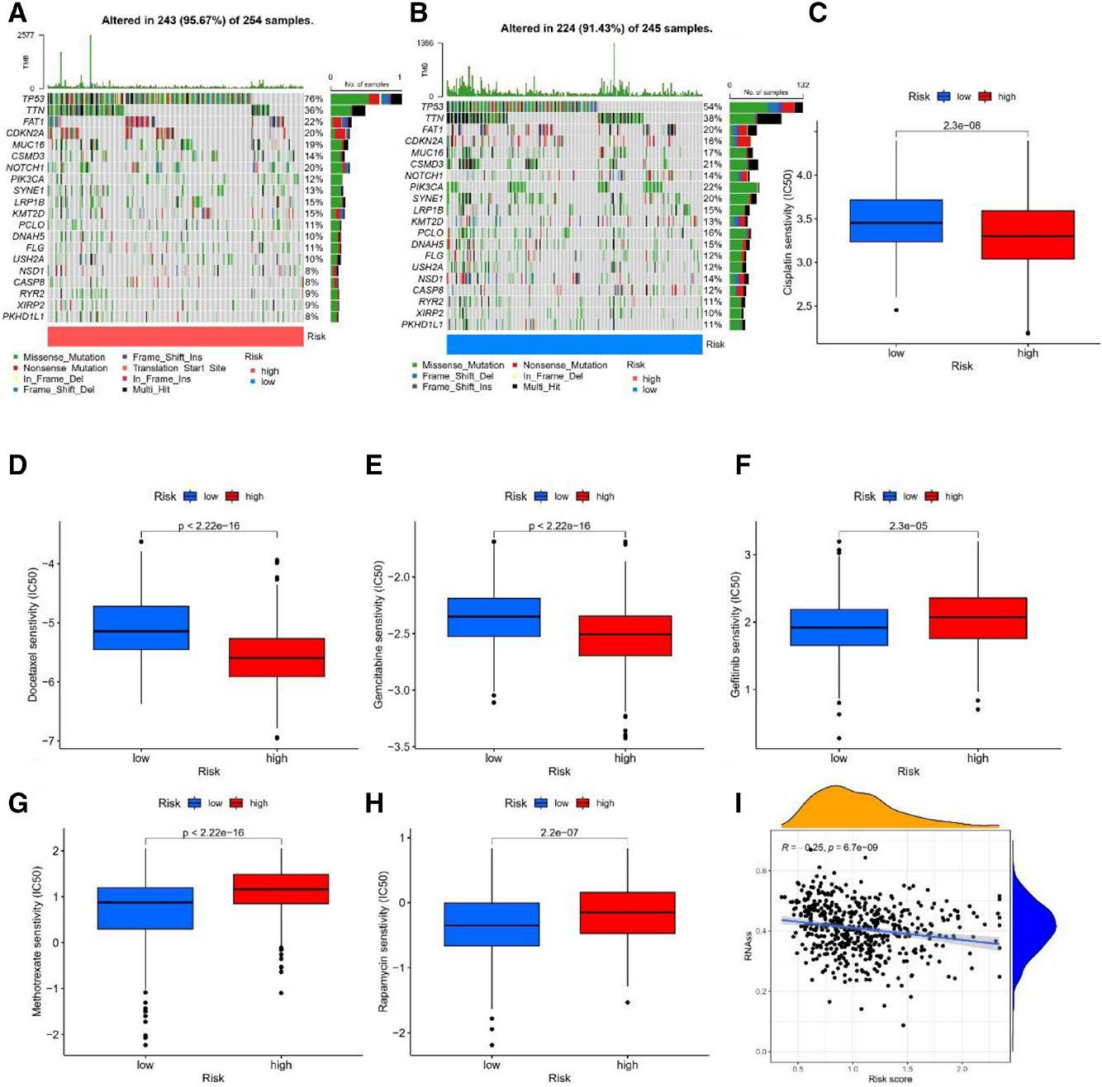
Mutation and drug sensitivity analysis. (A, B) Waterfall plots showing somatic mutational features in the disulfidptosis risk model. (C-H) Examples of disulfidptosis risk models aiding anti-tumor drug candidate selection. (I) Correlation between cancer stem cell index and DAG scores. DAGs = disulfidptosis-associated genes.

In Figure 8A and B, the upper bar shows the TMB, and we can observe that the TMB in the high subgroup population is lower than that in the low subgroup population. the top 20 genes with the highest mutation frequency were identified (Fig. 8A and B). The top 5 mutated genes were TP53, TTN, FAT1, CDKN2A, MUC16, in that order. We found that cisplatin, docetaxel, and gemcitabine had lower IC_50_ values in patients in the high disulfidptosis risk score group (Fig. 8C–E), whereas methotrexate, rapamycin, and gefitinib had lower IC_50_ values in patients with lower disulfidptosis risk scores (Fig. 8F–H). These results suggest that disulfidptosis risk models can help predict drug effectiveness in patients with HNSCC. Tumor stem cells are a class of cells with characteristics such as self-renewal, pluripotency, and tumor initiation that drive tumor growth and recurrence and are resistant to many current therapies. The DAG score and the Cancer Stem Cell Index were combined to assess the relationship between them. A slight but significant negative correlation was detected (*R* = −0.25, *P* = 6.7e-09) (Fig. 8I).

### 3.8. Constructing a disulfidptosis prediction line graph for HNSCC

**Figure 9.**
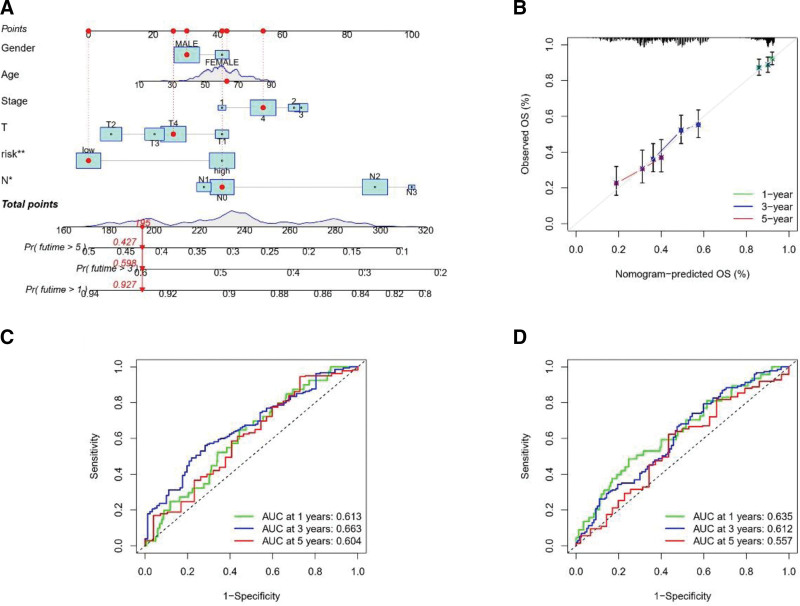
Constructing a disulfidptosis prediction line graph for HNSCC. Building a disulfidptosis predictive nomogram for HNSCC. (A) Disulfidptosis predictive nomogram predicted 1, 3, and 5 years OS in HNSCC patients. (B) Calibration curves for disulfidptosis predictive nomograms used to predict 1, 3, and 5 years OS in HNSCC patients. (C, D) ROC curves for disulfidptosis predictive nomogram used to predict 1, 3, and 5 years OS in HNSCC patients. P values were showed as: *, *P* < .05; **, *P* < .01; ***, *P* < .001. DAGs = disulfidptosis-associated genes, HNSCC = head and neck squamous cell carcinoma, ROC = receiver operating characteristic.

The disulfidptosis prediction column line plot was used to predict the probability of OS in patients with HNSCC, and the results showed that T-stage and disulfidptosis risk scores were independent prognostic factors (Fig. 9A). Calibration curves showed that the column line plot predicted 1-year, 3-year, and 5-year OS relatively well compared to the ideal model in the entire cohort (Fig. 9B). The AUC results showed that the 1-year, 3-year, and 5-year OS accuracy was more satisfactory for patients in both the training and test groups. The AUC values for the training group were 0.613, 0.663, and 0.604 at 1, 3, and 5 years, respectively (Fig. 9C). The AUC values for the test group were 0.635, 0.612, and 0.557 at 1, 3, and 5 years, respectively (Fig. 9D). All these findings suggest the advantage of our line graph in predicting the prognosis of HNSCC patients.

### 3.9. Validation of risk genes at methylation and total protein levels

**Figure 10.**
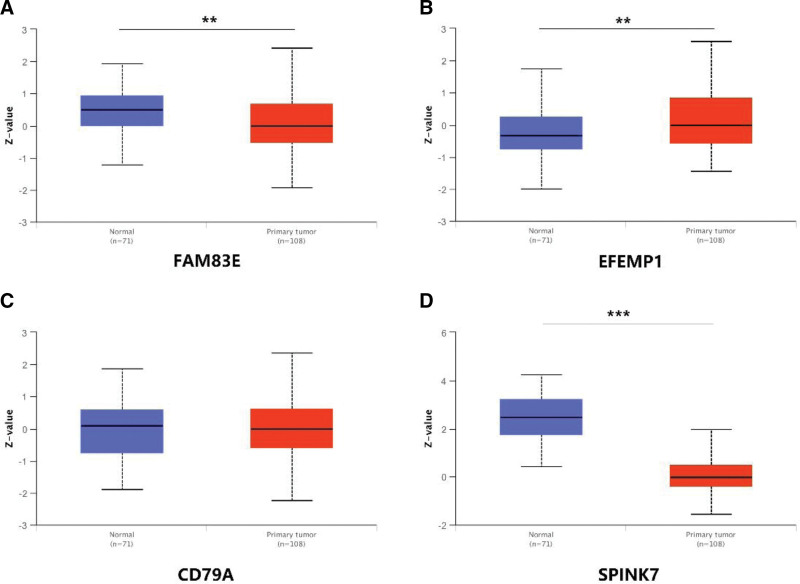
Total protein expression results of FAM83E, EFEMP1, CD79A, and SPINK7. (A–D) Total protein expression levels of FAM83E, EFEMP1, CD79A, and SPINK7 in HNSCC tissues. HNSCC = head and neck squamous cell carcinoma.

**Figure 11.**
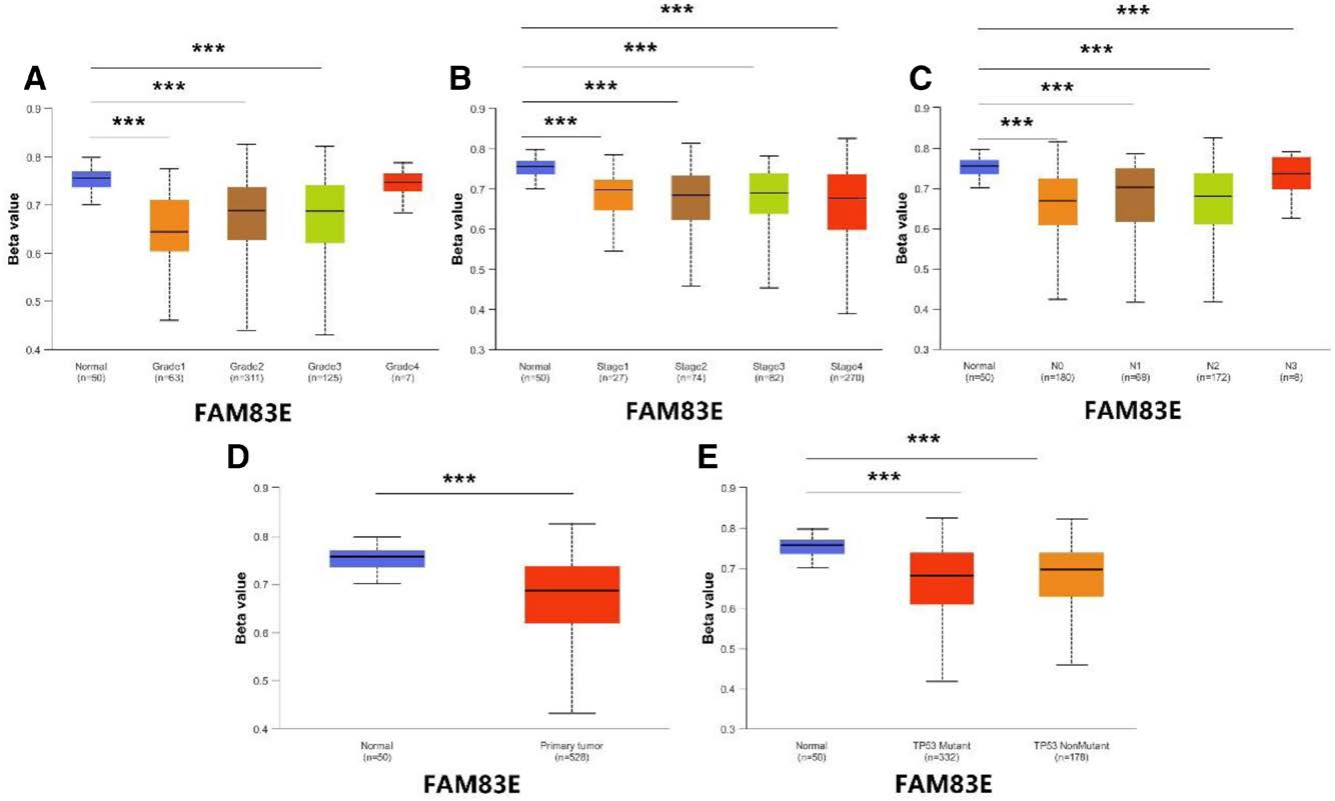
FAM83E methylation expression under different clinical characteristics. (A–C) Methylation levels of FAM83E under different TMN characteristics. (D) Methylation levels of FAM83E in different disease states. (E) Methylation levels of FAM83E between different TP53 mutation subgroups.

**Figure 12.**
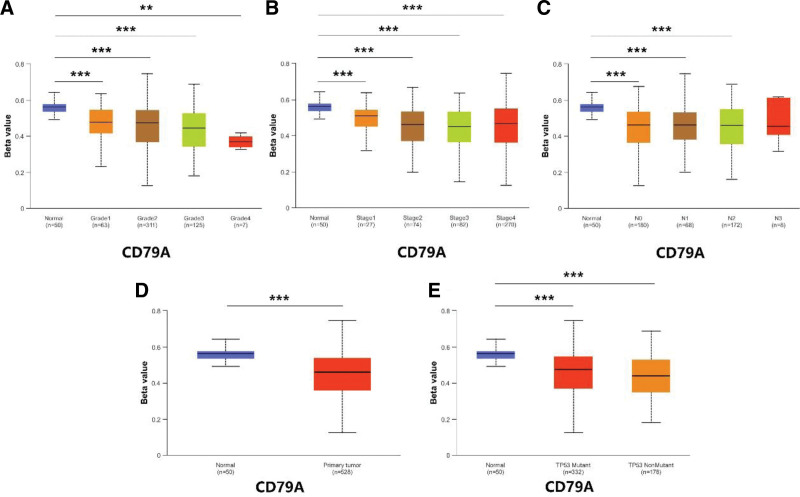
CD79A methylation expression under different clinical characteristics. (A–C) Methylation levels of CD79A under different TMN characteristics. (D) Methylation levels of CD79A in different disease states. (E) Methylation levels of CD79A between different TP53 mutation subgroups.

**Figure 13.**
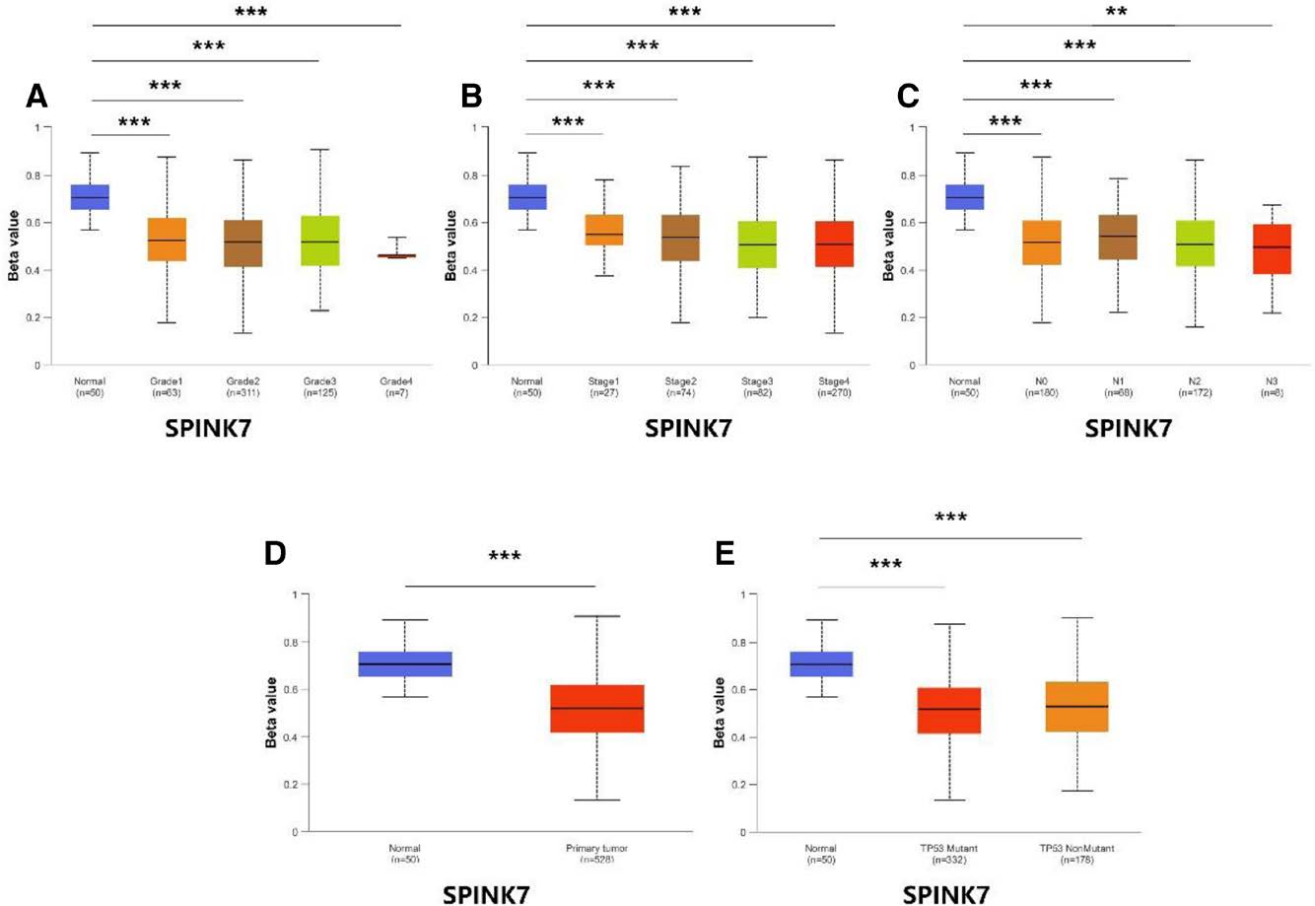
SPINK7 methylation expression under different clinical characteristics. (A–C) Methylation levels of SPINK7 under different TMN characteristics. (D) Methylation levels of SPINK7 in different disease states. (E) Methylation levels of SPINK7 between different TP53 mutation subgroups.

**Figure 14.**
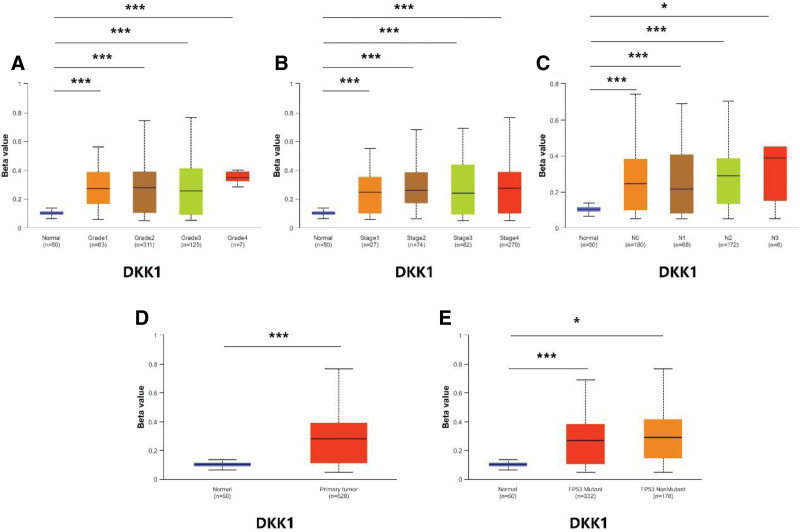
DKK1 methylation expression under different clinical characteristics. (A–C) Methylation levels of DKK1 under different TMN characteristics. (D) Methylation levels of DKK1 in different disease states. (E) Methylation levels of DKK1 between different TP53 mutation subgroups.

**Figure 15.**
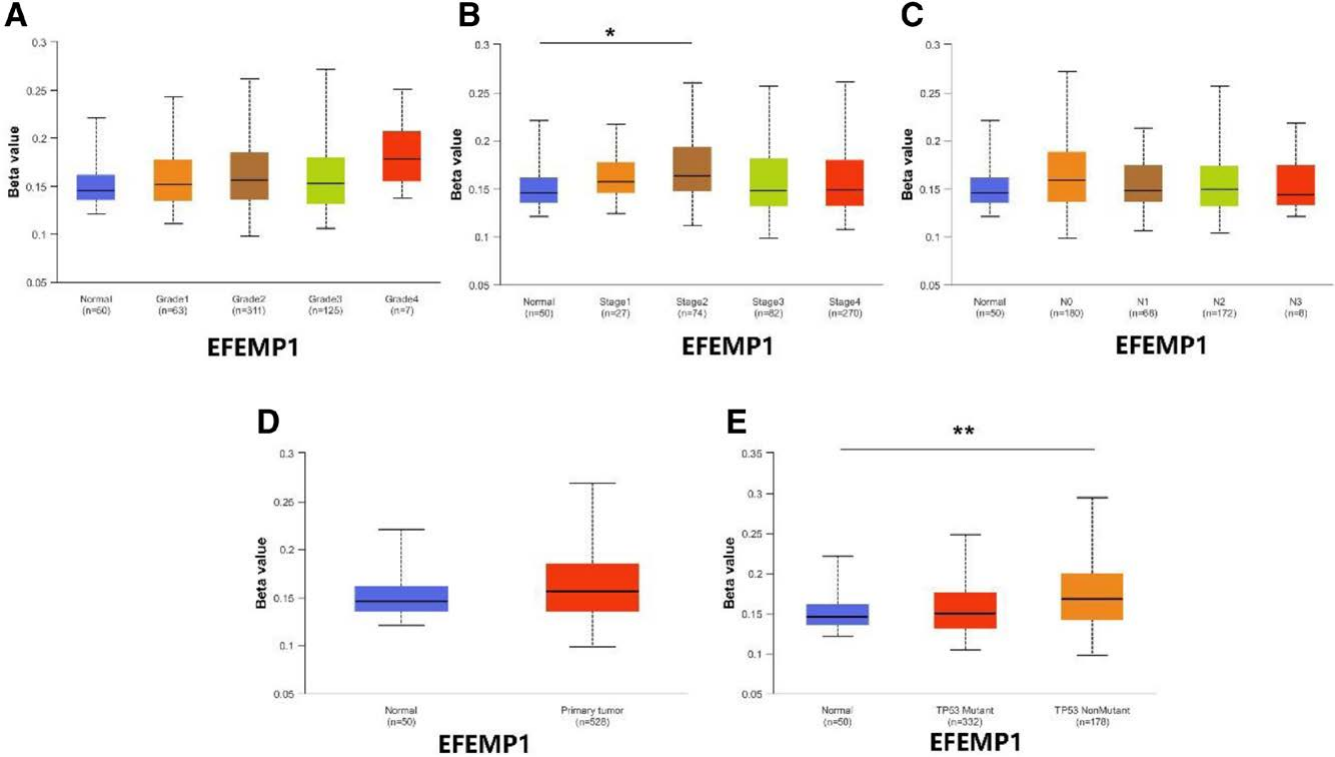
EFEMP1 methylation expression under different clinical characteristics. (A–C) Methylation levels of EFEMP1 under different TMN characteristics. (D) Methylation levels of EFEMP1 in different disease states. (E) Methylation levels of EFEMP1 between different TP53 mutation subgroups.

To further investigate the expression of risk genes in HNSCC, we downloaded the total protein expression levels of the corresponding risk genes in the UALCAN database to validate the results. The results showed that EFEMP1 was highly expressed in tumor tissues, FAM83E and SPINK7 were lowly expressed, and there was no significant difference in the expression level of CD79A (Fig. 10). Unfortunately, we did not find SPP2 in the UALCAN.

database to find the results of DKK1. In addition, we also downloaded methylation data of FAM83E, EFEMP1, CD79A, and SPINK7 in the UALCAN database. The results showed that the methylation levels of FAM83E (Fig. 11), CD79A (Fig. 12), and SPINK7 (Fig. 13) were reduced in tumor tissues with poor clinical staging, while DKK1 was elevated in tumor tissues (Fig. 14). There was no significant difference in the methylation levels of EFEMP1 (Fig. 15).

### 3.10. Tissue samples and quantitative real-time PCR

**Figure 16.**
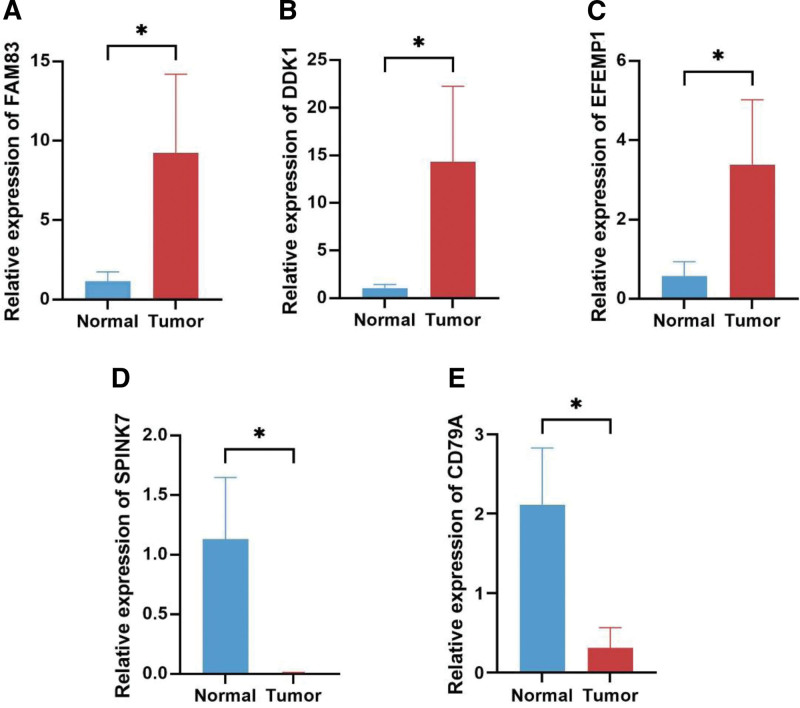
The Expression of 5 signature genes was verified by quantitative real-time PCR (qRT-PCR). (A–E) Expression of genes at the mRNA level by qRT-PCR.

To verify the expression levels of risk genes in HNSCC, we collected 4 cancerous and normal tissues from SYSUCC. As shown in Figure 16A, B, and D, qRT-PCR showed that the expression of FAM83 and DKK1 was significantly upregulated in tumor samples, while SPINK7 was downregulated. However, there was no significant difference in the expression of EFEMP1 and CD79A. Further studies showed heterogeneity in EFEMP1 and CD79A expression. Briefly, EFEMP1 was up-regulated in 3 patients (Fig. 16C) and CD79A was down-regulated in 3 patients (Fig. 16E).

## 4. Discussion

Head and neck cancer is the sixth most common cancer across the globe.^[9]^ They arise in the upper aerodigestive tract (including the oral cavity, pharynx, and larynx). HNSCC exhibits a high degree of heterogeneity and profound variation in treatment response.^[10]^ Because the head and neck are home to many vital organs that control important physiological functions, a large number of muscles, bones, blood vessels, and nerves are concentrated in a rather small space, and organ sites are interlocked,^[11]^ and conventional treatments such as surgery, chemotherapy, and radiation therapy are difficult to eradicate. TNM stage and histologic grade are closely related to the prognosis of head and neck squamous cell carcinoma and are the main basis and foundation for the selection of prognostic grading, immunotherapy, radiotherapy, and other treatment options.^[12]^ However, patients with HNSCC may exhibit different clinicopathological features, which suggests that the traditional clinicopathological staging may not be fully accurate.^[13]^ Therefore, the search for new prognostic biomarkers is crucial to improve the quality of life of patients with HNSCC.

Cell death is essential for maintaining homeostasis, development, and prevention of over proliferative malignancies in the body.^[14,15]^ A recent study showed that SLC7A11-mediated cystine uptake inhibits desmoplastic anemia but promotes cell death in response to glucose starvation, termed disulfidptosis. This is a form of cell death induced by the abnormal accumulation of high intracellular disulfides in SLC7A11 under glucose starvation conditions, which is distinct from apoptosis and demyelinating anemia.^[8]^ Disulfide polymerization in mitochondria can control cancer cell fate.^[16]^ However, the relationship between HNSCC and disulfidptosis remains unclear.

We first described the landscape of genetic and transcriptional variation in DAGs in HNSCC. We found that 12 DAGs were upregulated between HNSCC and normal tissues. GSVA analysis showed that DAG cluster A was significantly enriched in a variety of metabolism-related pathways, cluster B is highly expressed in cancer-related pathways. Disulfidptosis is associated with abnormal accumulation of intracellular disulfides. Patients were evaluated using disulfidptosis risk model and divided into high- and low-risk groups, with significant differences in prognosis between the 2 gene clusters. To investigate the reasons for the differences in prognosis, we performed GSVA analysis, which revealed the importance of the tumor’s metabolic microenvironment. KEGG results include signal transduction and cellular processes, among others, and these pathways correlate with the finding that high expression of SLC7A11 combined with glucose starvation causes disulfide death by disulfide stress.^[8]^ These results suggest that DAGs regulate the tumor microenvironment and influence the development of head and neck squamous carcinoma.

Disulfide is important for cellular immunity. According to reports, Disulfiram enhances T-cell anti-tumor immunity by directly activating lymphocyte-specific protein tyrosine kinase (LCK)-mediated TCR signaling.^[17]^ And diflunisal targets HMGB1/CXCL12 heterotrimeric complex and blocks immune cell recruitment.^[18]^ We evaluated the association between disulfidptosis risk model and immune cell abundance. The results showed that most immune cells were significantly associated with DAG and risk genes, risk scores were positively correlated with macrophage M0 and negatively correlated with T cells CD4 + memory activated, plasma cells, and regulatory T cells (Tregs). In cancer, tumor-associated macrophages can suppress anti-tumor immunity, promote tumor progression, and negatively correlate with patient prognosis.^[19]^ T cells CD4 + memory activated, plasma cells were associated with longer OS.^[20]^ Our results of reduced T cell CD4 + memory activation and plasma cell abundance and poor prognosis in the high-risk group, as opposed to macrophages, are consistent with previous findings. Central memory CD4 + T cells maintain immune memory and play an immunoprotective role during tumor metastasis.^[21,22]^ Effector memory CD4^+^ T cells express adhesion molecules and chemokine receptors that perform rapid functions.^[23,24]^ Studies have reported that patients with high memory T-cell activation have shorter OS, whereas those with high memory T-cell quiescence have longer OS.^[25]^ However, a higher proportion of Tregs was found in the low-risk score group than in the high-risk score group. One possible reason for this discrepancy is caused by glucose starvation in disulfidptosis. In colon cancer, 2 Treg cell subtypes were shown to play opposite functions in regulating the tumor microenvironment.^[26]^

DNA hypermethylation is closely associated with the development of cancer. Specifically, some oncogene copies are hypermethylated or naturally mutated.^[27]^ To fully understand the correlation between the methylation levels of risk genes and clinical features, we analyzed the expression levels of risk genes under different clinical features. The results showed that hypermethylation was associated with poor clinical prognosis in HNSCC, consistent with previous findings.^[28]^ To verify the protein expression of risk genes in HNSCC tissues, we visualized risk genes using the UALCAN database. The results showed that the expression of EFEMPP1 was up-regulated in HNSCC tissues, whereas the expression of FAM83E and SPINK7 was down-regulated in HNSCC tissues, and there was no significant difference in the expression level of CD79A. Unfortunately, we did not find data related to DKK1, which may be the reason for the low level of DKK1 as a secreted protein in HNSCC tissues.

Among the risk genes, DDK1 and EFEMP1 had a higher risk factor. The pro-oncogenic role of DKK1 in prostate cancer (PCa) bone metastases has been reported to be associated with increased growth of bone metastatic foci, decreased osteoinduction, and altered signaling through a typical pathway that is not dependent on WNT.DKK-1 may be a promising therapeutic target for PCa.^[29]^ There is evidence that EFEMP1 is associated with tumor aggressiveness and metastasis.^[30]^ FAM83E may regulate or be regulated by KRAS or SMAD4 in pancreatic ductal adenocarcinoma, leading to cancer progression.^[31]^ The expression changes of SPINK7 can be relevant in predicting OSCC at a molecular level.^[32]^

Considering the impact of disulfidptosis on HNSCC heterogeneity and corresponding clinical outcomes, we constructed a disulfidptosis risk model based on 5 risk genes and a column line graph to quantify the risk score. These results suggest that the disulfidptosis risk model score can be used as an independent prognostic biomarker for HNSCC patients. We performed antitumor drug sensitivity analysis on different populations, which allowed the disulfidptosis risk model to predict prognosis and drug candidates. The benefits of using risk scores and clinical characteristics can help physicians understand the current and future status of their patients. This study has several limitations. First, the sample in this study was limited, and further cohort studies are needed to validate the risk assessment model. Second, we performed a complete bioinformatics study without further investigation of the mechanism of target genes on HNSCC, so basic experiments are necessary for future researchers to further explore the mechanism. Besides there are still unanswered questions about the mechanism of carbon disulfide poisoning. In addition, we investigated only a few of the most promising markers in this study, and additional genes could be included in the future to construct a more accurate prognostic model. In summary, we conducted a comprehensive analysis of disulfidptosis-associated genes, including their impact on the tumor’s metabolic and immunological microenvironment, clinicopathological features, and prognosis. We have developed a disulfidptosis risk model and performed sensitivity analysis on anticancer drugs. Our findings can be used to design individualized treatment strategies for patients with different subtypes of HNSCC.

## Tissue samples

HNSCC specimens were obtained from cancer patients, while normal tissue specimens were obtained from paracancerous tissues. Informed consent forms were completed by all patients. Samples were promptly frozen at −80°C upon collection and were only thawed 10 minutes before any biochemical analysis was conducted. The study was approved by the Ethics Committee of the Hospital of Stomatology, Jilin University.

Furthermore, risk genes promoter methylation and total protein data were downloaded from the UALCAN database.

## Author contributions

**Conceptualization:** Yushen Li.

**Data curation:** Yushen Li, Lu Tao, Jiajun Xin, Bowei Wang.

**Formal analysis:** Yushen Li, Lu Tao, Xiantao Chen, Rui Wang, Bowei Wang, Zhihui Liu.

**Funding acquisition:** Bowei Wang, Zhihui Liu.

**Investigation:** Yushen Li, Xiantao Chen, Rui Wang, Bowei Wang.

**Methodology:** Yushen Li, Lu Tao, Yifei Dai, Bowei Wang.

**Project administration:** Yifei Dai, Zhihui Liu.

**Resources:** Jiatong Zou, Zhihui Liu.

**Software:** Yushen Li, Jiajun Xin, Bowei Wang.

**Supervision:** Yushen Li, Lu Tao, Jiatong Zou, Bowei Wang.

**Validation:** Yushen Li, Lu Tao, Bowei Wang.

**Visualization:** Yushen Li, Zhihui Liu.

**Writing – original draft:** Yushen Li, Zhihui Liu.
